# Polarization and incident angle independent multifunctional tunable terahertz metasurface based on graphene

**DOI:** 10.1038/s41598-024-55676-4

**Published:** 2024-03-01

**Authors:** Ubaid Ur Rahman Qureshi, Shahid Basir, Fatma Mallek, Habib Hamam

**Affiliations:** 1 Beijing Engineering Research Center for Mixed Reality and Advanced Display, School of Optics and Photonics, Beijing Institute of Technology, Beijing, 100081 China; 2 School of Engineering and Applied Sciences, ISRA University, Islamabad, Pakistan; 3 Faculty of Engineering, Uni de Moncton, Moncton, NB E1A3E9 Canada; 4 International Institute of Technology and Management (IITG), Avenue des Grandes Ecoles, Libreville, Gabon; 5 Department of Electrical and Electronic Engineering Science, School of Electrical Engineering, University of Johannesburg, Johannesburg, 2006 South Africa

**Keywords:** Energy science and technology, Materials science, Nanoscience and technology, Optics and photonics, Physics

## Abstract

Motivated by the imperative demand for design integration and miniaturization within the terahertz (THz) spectrum, this study presents an innovative solution to the challenges associated with singular functionality, limited application scope, and intricate structures prevalent in conventional metasurfaces. The proposed multifunctional tunable metasurface leverages a hybridized grapheme–metal structure, addressing critical limitations in existing designs. Comprising three distinct layers, namely a graphene–gold resonance layer, a Topas dielectric layer, and a bottom gold film reflective layer, this terahertz metasurface exhibits multifunctionality that is both polarization and incident-angle independent. The metasurface demonstrates a broadband circular dichroism (CD) function when subjected to incident circularly polarized waves. In contrast, under linear incidence, the proposed design achieves functionalities encompassing linear dichroism (LD) and polarization conversion. Remarkably, graphene's chemical potential and the incident light’s state can be manipulated to tune each functional aspect's intensity finely. The proposed tunable multifaceted metasurface showcases significant referential importance within the terahertz spectrum, mainly contributing to advancing CD metamirrors, chiral photodetectors, polarization digital imaging systems, and intelligent switches.

## Introduction

The burgeoning advancement in Terahertz (THz) science and technology, encompassing areas such as satellite communication, spectroscopic detection, and sensing, has captivated widespread interest^1–5^. Controlling the propagation of THz waves conventionally hinges on the accumulation of phase, a process that poses notable challenges when attempting the direct interaction of THz radiation with materials found in nature^6^. To surmount this inherent constraint, metasurface have arisen as an exceptionally efficient solution.

Metasurface, which consist of systematically organized artificial units smaller than the wavelength of light, have attracted increasing attention due to their considerable potential for use in perfect absorbers^7,8^, circular dichroism (CD)^9,10^, and polarization converters^11–13^. Furthermore, they display unique electromagnetic effects and innovative physical characteristics. However, metasurface composed of traditional metals cannot sometimes modify their functionality after manufacture, which presents difficulties for complex real-world applications. Hence, exploring tunable and versatile metasurface has been a critical focus of research in photonic devices. Graphene, a material capable of undergoing phase shift, shows great potential for creating tunable devices operating in the THz and mid-infrared spectral ranges. The optical properties of graphene depend on its surface conductivity, which can be carefully adjusted by applying external bias voltages. Graphene's remarkable electrical and optical characteristics have resulted in its widespread application in several fields, such as absorbers^14,15^, wavefront modification^16^, sensors^17,18^, antennas^19,20^, and demultiplexers^21^. Researchers have investigated dichroism, a crucial component of terahertz metasurface applications^22,23^. Dichroism pertains to the absorption characteristics of a material contingent upon the polarization of the incident electromagnetic (EM) waves, specifically those orthogonally polarized. Dichroism manifests in two distinct modalities, namely circular dichroism (CD) and linear dichroism (LD). The term "CD" describes the difference in chiral metamaterials' absorption under two different rotationally circularly polarized (CP) light sources^24–26^. The difference in metamaterial absorption upon incident to transverse magnetic (TM) and transverse electric (TE) polarization waves is known as linear dichroism (LD)^27,28^. Natural dichroic materials, such as dichroic crystals, DNA, and proteins, exhibit weak dichroic responses that require considerable interaction paths for observation^29–31^. This drawback has prompted the development of metasurfaces as efficient alternatives. Metasurfaces possess strong light-matter interactions that can overcome weak dichroism and achieve distinct LD and CD responses. Recently, reflective metasurfaces have demonstrated strong anisotropic responses, enabling the realization of LD and CD with high efficiency^32–37^. However, current metasurfaces have limited functionality and face challenges such as narrow bandwidth, non-tunability, low efficiency, and complex control methods. Moreover, the process by which electromagnetic waves switch between two polarization modes is called polarization conversion. Prior investigations^13,38^ suggest that THz metasurfaces have been the focal point of considerable scholarly scrutiny. Presently, scholarly efforts are dedicated to investigating the amalgamation of diverse electromagnetic functionalities within a singular device^39,40^. This pursuit aims to efficiently mitigate the manufacturing expenses associated with metasurface and broaden their scope of applications. Consequently, numerous investigators have incorporated tunable materials into metasurface designs, coupled with a spectrum of active control methodologies encompassing optical excitation, mechanical stretching, thermal modulation, and external electric fields. These endeavors establish favorable conditions for the advancement of tunable multifunctional metasurface^41–45^.

In their work^41^, Xie et al. introduced a versatile metasurface that incorporates graphene and VO2, functioning as a broadband absorber, half-wave plate (HWP), and quarter-wave plate (QWP) across the terahertz frequency range. WEN et al.^42^ proposed and validated a dual-function metasurface employing gold nanorods, demonstrating holography and lens-switching capabilities by manipulating electromagnetic wave wavelength and polarization state. Furthermore^43^, presents an experimentally verified metamaterial device based on GST, showcasing cross-polarization conversion (CPC) in reflection mode and circular-to-linear polarization conversion in transmission mode under crystalline and amorphous GST conditions. Ghosh et al.^44^ reported a graphene-based terahertz metasurface functioning as a wideband tunable absorber with electromagnetic transmissivity. Furthermore, Qureshi et al.^45^ proposed a VO_2_-based metasurface exhibiting diverse THz functionalities, including perfect absorption, CD response for reflected and transmitted fields and electromagnetically induced transparency (EIT) effect, achieved through modulation of VO_2_ layer conductivity. Nevertheless, literature is needed regarding the existence of three or more tunable multifunctional metasurface. Furthermore, their designs and control techniques are frequently intricate, presenting substantial obstacles to practical production. Furthermore, metasurface functional devices currently need help with issues such as suboptimal efficiency, limited bandwidth, lack of tunability, and the need for intricate control mechanisms. Hence, our objective is to create a versatile metasurface that has a straightforward design and exceptional functionality. This will be advantageous in the development of THz photonic devices.

We propose a THz switchable metasurface independent of polarization and incident angle, employing a graphene–metal hybrid structure. Utilizing materials with significant differences in chemical potential, our design facilitates the excitation of the electromagnetic response to spin-selective incident light. The metasurface achieves versatile switching functionality by controlling electromagnetic wave incident and polarization angles. Theoretical outcomes indicate that under CP light incidence, the metasurface attains a CD intensity exceeding 40% within the 3.1 to 7.4 THz range, reaching a peak efficiency of CD 75% at 5.4 THz. Furthermore, under linearly polarized (LP) light incidence, the metasurface exhibits a LD intensity surpassing 60% within the 2.9 to 3.8 THz range, with a maximum efficiency of LD at 74% at 3.3 THz. Additionally, the metasurface achieves polarization conversion, converting linear and circular polarizations to their respective cross-polarizations in the 4.1 to 7.1 THz range, with a polarization conversion ratio reaching 100%. In contrast to prevalent multifunctional devices, our approach leverages the attributes of phase change materials (PCM) and electromagnetic wave polarization angles, minimizing structural complexity and integration challenges in multifunctional devices. This approach provides a novel avenue for the integration of photonic devices.

## Structure design and simulation method

A myriad of functionalities can be derived from a singular geometric configuration through meticulous structural design, thereby eliciting distinctive responses. Intricately crafted by incorporating a graphene–metal ring resonator, the proposed structure demonstrates multifunctional attributes. This topological configuration can be tailored to manifest CD, LD, and CPC effects by adeptly leveraging the distinctive properties of PCM and the polarization angle inherent in EM waves.

**Figure 1:**
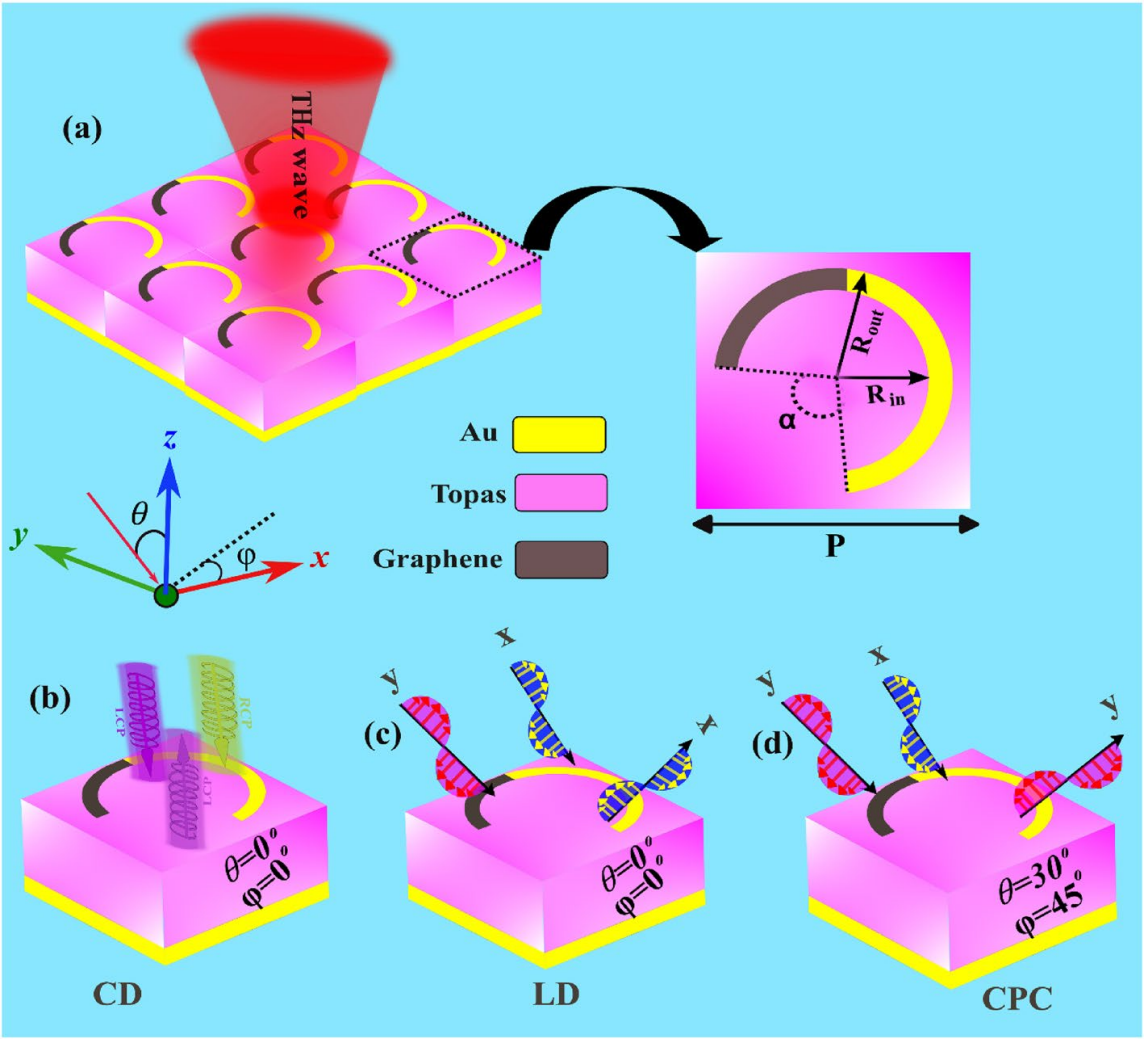
Schematic diagram of the proposed multifunctional tunable metasurface (**a**) Two-dimensional array of the unit cells. (**b**) Schematic diagrams show of the circular dichroism function; (**c**) Schematic diagrams show of the linear dichroism function (**d**) Schematic show of the polarization conversion functions.

The selection of the graphene–metal ring resonator structure as the fundamental unit cell for the metasurface enables the execution of various operations. The schematic representation of the metasurface is delineated in Fig. 1, comprising three stratified layers arranged from top to bottom: a graphene–gold resonance layer, a dielectric layer composed of polyethene cyclic olefin copolymer (Topas), and a reflective layer of the bottom gold film. The thickness of the graphene, gold, and Topas dielectric layer is reported to be 1 nm, 0.2 µm, and 11 µm, respectively. Notably, the symmetrical pattern of the graphene–metal split ring resonant layer is presented at 45° in the *xy* plane. Following parametric scanning, optimal geometric parameters are determined as P = 20 μm, Rin = 7 μm, Rout = 8.5 μm, and α = 100 μm, as depicted in Fig. 1a. The metallic layer is constructed from gold with a conductivity of σ = 4.07 × 10^7^ S/m. The dielectric layer employs Topa's material, characterized by a dielectric constant 2.35. This material exhibits transparency, thermal stability, exceptional optical properties, and a negligible absorption coefficient within the THz frequency band^46,47^.

In contrast to graphene-only structures, we used a graphene–gold ring resonator design. This unique design allows us to selectively regulate efficiency for CP and LP incident waves by applying a gate voltage to graphene and changing electromagnetic wave polarization.

The conductivity of graphene can be characterized by considering the combined effects of electron–photon scattering in both intra-band and inter-band transitions^48^:

1 $${\sigma }_{intra}=-\frac{j{e}^{2}{k}_{B}T}{\pi \hslash \left(\omega +j2\Gamma \right)}\left\{\frac{{E}_{f}}{{k}_{B}T}+2ln\left[1+{\text{exp}}\left(\frac{-{E}_{f}}{{k}_{B}T}\right)\right]\right\}$$

2 $${\sigma }_{inter}=-\frac{j{e}^{2}}{4\pi \mathrm{\hslash }}{\text{ln}}\left[\frac{2{E}_{f}-\left(\omega +j4\pi \Gamma \right)\mathrm{\hslash }}{2{E}_{f}+(\omega +j4\pi\Gamma )\mathrm{\hslash }}\right]$$

The symbols $\tau$, $\Gamma$*, *$\omega$*, *$T$*,* ${k}_{B}$ and $e$ denote the relaxation time, scattering coefficient, angular frequency of light, ambient temperature, Boltzmann constant and elementary charge, respectively. The terms $\mathrm{\hslash }$ and ${{\text{E}}}_{{\text{f}}}$ represent the reduced Planck's constant (commonly referred to as Dirac's constant) and chemical potential associated with charge carriers, respectively. In the THz frequency range, intra-band transitions exert a substantial influence. Consequently, the determination of graphene's surface conductivity in this frequency regime is approached through the application of the Drude model^49^.

3 $$\sigma =\frac{j{\mathcal{e}}^{2}{E}_{f}}{\pi \hslash (\omega +j{\tau }^{-1})}$$

The potential operational modes of the proposed design are delineated in Fig. 1b–d. Under incident CP light, the metasurface exhibits a broadband CD function, as depicted in Fig. 1b. Conversely, the metasurface demonstrates LD functionality when subjected to LP light, illustrated in Fig. 1c. Additionally, for specific angles $\theta =30^\circ$ and $\varphi =45^\circ$, as presented in Fig. 1d, the design realizes polarized conversion capabilities for LP and CP incident waves.

## Metasurface performance

### Circular dichroism

In order to obtain the desired CD response in the metasurface, it is essential to disturb the symmetries of the mirror and n-fold rotations (where n > 2) within the structure^13^. Figure 1 depicts a split ring resonator (SRR) that has been purposefully simulated using graphene and gold materials to amplify the interaction between incident waves and graphene while simultaneously removing any symmetries as the chemical potential of graphene increases. To precisely optimize the geometric characteristics, the commercial software CST (Microwave Studio) was applied.

The metasurface, featuring a metallic backplane, manifests null transmission coefficients, rendering an exhaustive analysis of its reflective attributes satisfactory. In adherence to the tenets of polarization optics, the intricacies of reflected LP waves can be correlated with incident LP waves through the application of the Jones matrix^44,45^:

4 $$\left(\genfrac{}{}{0pt}{}{{E}_{r}^{x}}{{E}_{r}^{y}}\right)=\left(\genfrac{}{}{0pt}{}{{R}_{xx}}{{R}_{yx}}\genfrac{}{}{0pt}{}{{ R}_{xy}}{{R}_{yy}}\right)\left(\genfrac{}{}{0pt}{}{{E}_{i}^{x}}{{E}_{i}^{y}}\right)={\varvec{R}}\left(\genfrac{}{}{0pt}{}{{E}_{i}^{x}}{{E}_{i}^{y}}\right)$$

The reflection matrix, denoted as R, encapsulates the Cartesian Jones reflection matrix attributes intrinsic to the metasurface. This matrix is employed in conjunction with the incident electric field ${E}_{i}^{x(y)}$​ and the reflected electric field ${E}_{r}^{x(y)}$​, both aligned in the $x(y)$ direction. The derivation of the reflection matrix concerning CP states is achieved through a coordinate transformation from the Cartesian basis to the circular basis, utilizing LP reflection coefficients.

5 $$\begin{aligned} \varvec{R}_{{\varvec{CP}}} & = \left( {\begin{array}{*{20}l} {R_{{ + + }} } & {R_{{ + - }} } \\ {R_{{ - + }} } & {R_{{ - - }} } \\ \end{array} } \right) = \varvec{\Lambda }^{{ - 1}} \varvec{R\Lambda } \\ & = \frac{1}{2}\left( {\begin{array}{*{20}l} {(R_{{xx}} ) - (R_{{yy}} ) - i\left( {R_{{xy}} + R_{{yx}} } \right)} & {(R_{{xx}} ) + \left( {R_{{yy}} } \right) + i\left( {R_{{xy}} - R_{{yx}} } \right)} \\ {\left( {R_{{xx}} } \right) + \left( {R_{{yy}} } \right) - i\left( {R_{{xy}} - R_{{yx}} } \right)} & {(R_{{xx}} ) - \left( {R_{{yy}} } \right) + i\left( {R_{{xy}} + R_{{yx}} } \right)} \\ \end{array} } \right) \\ \end{aligned}$$

The coordinate transformation matrix denoted as Λ, characterized by $\Lambda =\frac{1}{\sqrt{2}}\left(\genfrac{}{}{0pt}{}{1}{i} \genfrac{}{}{0pt}{}{1}{-i}\right)$, is employed to facilitate the conversion from Cartesian base to circular base. Within this framework, the symbols "+" and "−" are employed to signify clockwise and counter-clockwise CP waves, respectively. Furthermore, "$x$" and "$y$" denote the components associated with *x*-polarized and *y*-polarized vectors.

The formula for calculating CD is as follows^50^:

6 $${\text{CD}}={{\text{A}}}_{+}-{{\text{A}}}_{-}$$

7 $${{\text{A}}}_{-}^{ }=1-({{\text{R}}}_{+-})-({{\text{R}}}_{-})$$

8 $${A}_{+}=1-({R}_{-+})-({R}_{++})$$

The absorptions of LCP and RCP waves are denoted by A_−_ and A_+_, respectively, with their disparity representing the CD. Electromagnetic waves spanning a frequency range from 1 to 9 THz were incident upon the metasurface via two ports, from which the ensuing reflection coefficients were derived.

**Figure 2:**
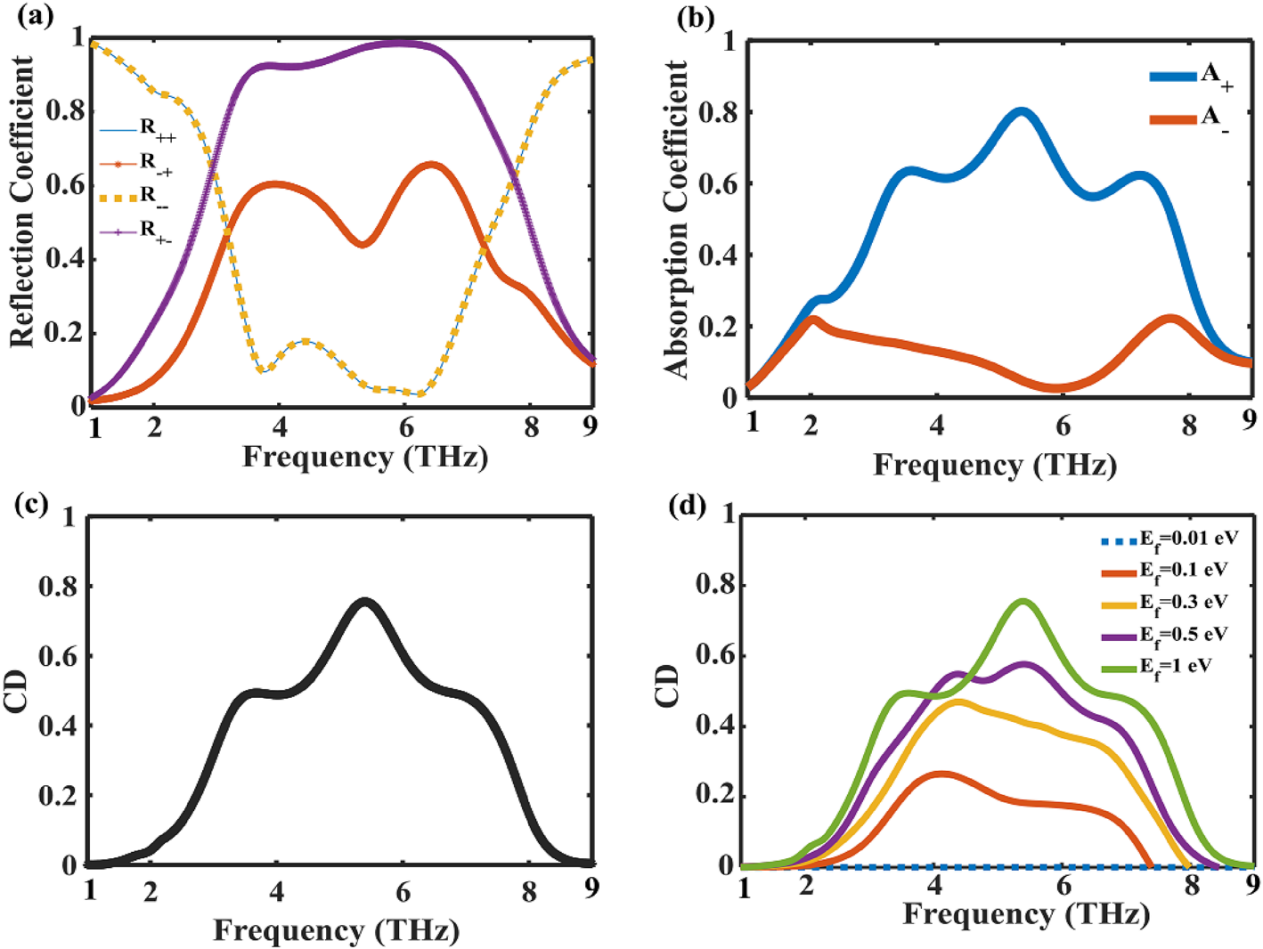
(**a**) Reflection coefficient of the graphene-based metasurface at a Fermi level of E_f_ = 1 eV under the excitations of a CP normally incident wave. (**b**) Absorption coefficient of the proposed design under a normally incident wave. (**c**) CD spectra of the proposed design. (**d**) CD of the Proposed Structure for Various Chemical Potentials.

The reflection coefficients for RCP and LCP waves are shown in Fig. 2a. The reflection spectra distinctly exhibit resonance regions within ultrawideband (3.1–7.4 THz) when the CP waves are normally incident at fermi level of 1 eV, as depicted in Fig. 2a. A noteworthy distinction arises in the conversion reflection between RCP (${R}_{-+}$) waves and LCP (${R}_{+-}$) waves, attributable to the pronounced chirality exhibited by the metasurface. This inherent chiral behavior serves as the driving force behind the prominently observed and highly influential CD effect within our proposed structure.

The absorption spectra exhibit wideband resonance regions within 3.1–7.4 THz, where the absorptions of RCP and LCP waves (A+ and A−) display notable differences under the normal incidence of circularly polarized waves, with fermi levels of 1 eV, as illustrated in Fig. 2b. At 5.4 THz, the absorption rates for RCP incident waves reach approximately 80%, indicating good absorption. The CD values achieved are more than 40% in 3.1–7.4 THz and reach 75% at 5.4 THz as shown in Fig. 2c. The manifestation of CD is ascribed to the profound interaction between electric and magnetic dipoles. The disruption of mirror symmetry induces alterations in dipole interactions, leading to electromagnetic responses characterized by Fano resonances.

The ability to control CD spectra has significant advantages in biosensing applications due to the inherent chirality of biomolecules such as DNA and proteins. The proposed device allows for tunable and controllable CD spectra by manipulating graphene's external electrical bias voltage. This eliminates the necessity for redesigning or employing intricate equipment such as pump lasers. To examine this ability, we methodically raised the fermi level of graphene from 0.01 to 1 eV and calculated the CD, as depicted in Fig. 2d. According to Fig. 2d, when the chemical potential of graphene varies from 0.01 to 1 eV, the CD value may be controlled from 0 to a maximum of 0.75. This means the CD metamirror can switch between the "on" and "off" states. Furthermore, we determine the modulation depth (MD)^51^ of CD using the subsequent equation:

9 $$MD=\frac{C{D}_{max}-C{D}_{min}}{C{D}_{max}}$$

The MD value is determined to be greater than 0.98 within the frequency range of 3.1 to 7.4 THz. Therefore, the proposed structure is very suitable for application in modulation technology.

**Figure 3:**
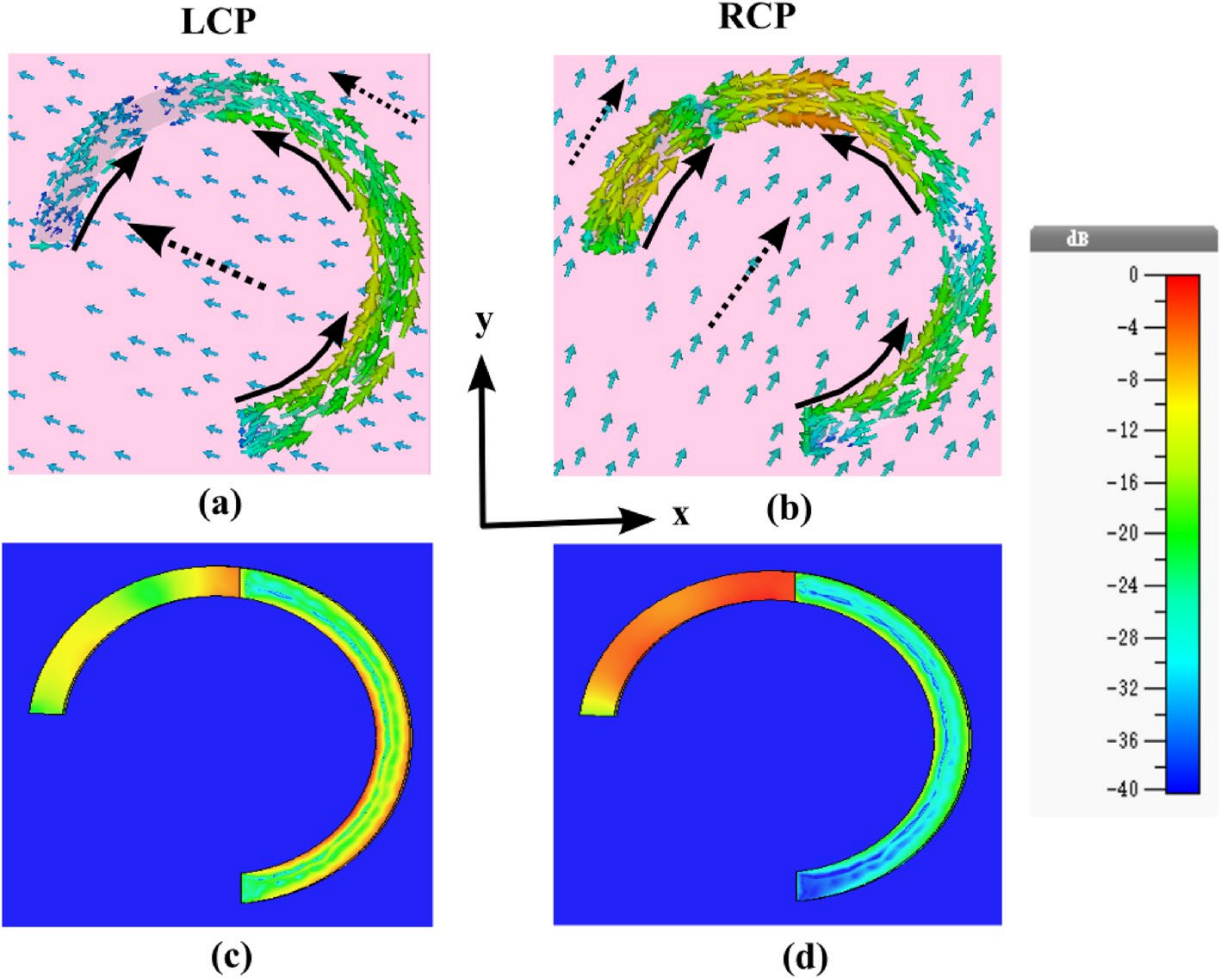
(**a**,**b**) Surface current distributions at a Fermi level of E_f_ = 1 eV when stimulated by LCP and RCP incident waves at a frequency of 5.4 THz. Solid line arrows depict the front view of the surface, while solid dashed arrows denote the surface current distributions on the rear side. (**c**,**d**) Power loss density patterns at a Fermi level of E_f_ = 1 eV when stimulated by LCP and RCP incident waves at a frequency of 5.4 THz.

A comprehensive scrutiny of surface current and power loss density distributions at the resonance frequency dip (5.4 THz) was conducted to elucidate the fundamental physical mechanisms governing the phenomenon of selective absorption in a chiral metasurface. Upon the incidence of CP waves onto the proposed metasurface, examining surface current distributions at various resonance frequencies revealed antiparallel orientations on the upper and lower surfaces of the unit cell, as delineated in Fig. 3. This spatial configuration mitigated the virtual magnetic response, thereby instigating the emergence of electric and magnetic dipoles oriented along specific axes. Subsequently, a conspicuous cross-coupling between electric and magnetic fields ensued, amplifying the chirality effect and augmenting chiral-selective absorption^45,52^. Each resonance frequency arises from the amalgamation of these discrete electric and magnetic response patterns within the envisioned chiral structure.

Notably, under the normal incidence of LCP waves, the induced surface current distributions on the top and bottom surfaces of the unit cell exhibit parallel and antiparallel configurations, respectively, indicative of a feeble magnetic response mode associated with diminished absorption, as illustrated in Fig. 3a. In contrast, for RCP incident waves, the induced surface current distributions assume antiparallel orientations, signifying a robust magnetic response mode that begets heightened absorption, as depicted in Fig. 3b.

Further scrutiny of the proposed unit cell structure involves simulating power loss density distribution generated by distinct CP waves at 5.4 THz. This analysis furnishes additional insights into the fundamental physical mechanism of chiral-selective resonant absorption within the contemplated chiral metasurface, as illustrated in Fig. 3c and d. The observed THz wave absorption manifests across discrete sections of the chiral structure, delineating the intricate interplay between distinct CP waves and the structural components. Notably, the symmetrical pattern of the graphene–metal split ring resonant layer is presented at 45° in the *xoy* plane, which interplay gives rise to varied chiral currents within the unit cell and, consequently, divergent deep absorptions. Precisely, for LCP waves, a subdued resonant absorption is discerned at 5.4 THz (Fig. 3c), while for RCP waves, a conspicuous resonant absorption is evident at the same frequency (Fig. 3d).

**Figure 4:**
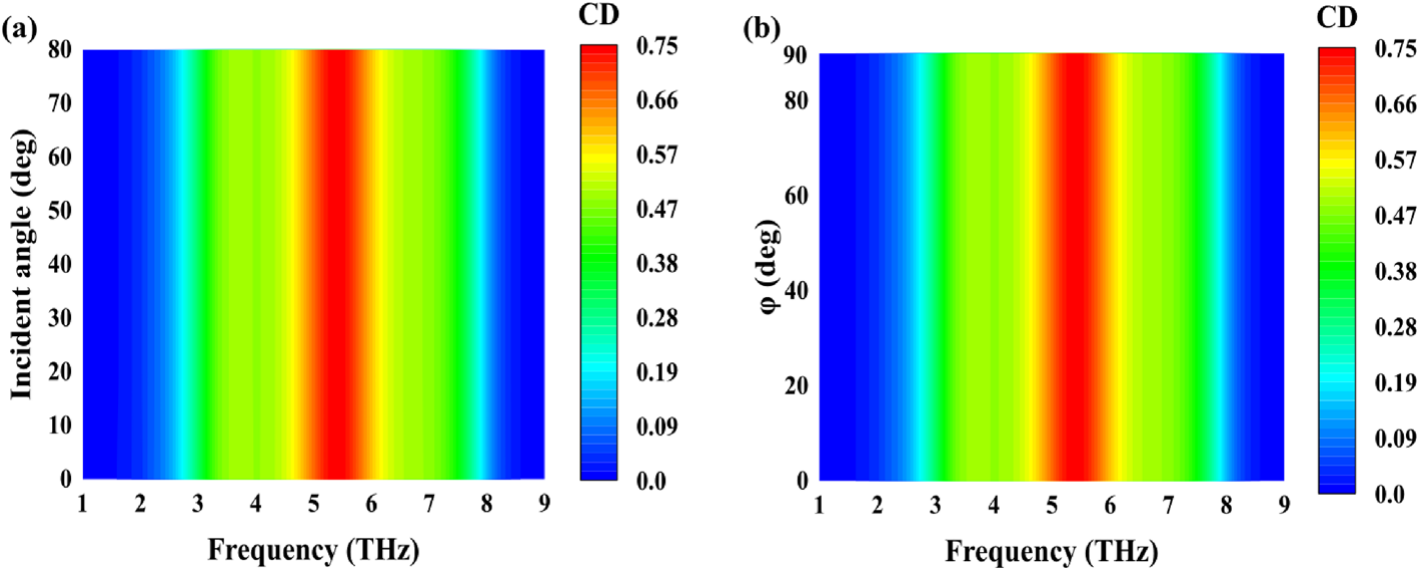
(**a**) CD of the proposed structure at a Fermi level of E_f_ = 1 eV at different incident angle (**b**) Simulated CD at various polarization angles.

In practical applications, evaluating metasurface performance often involves considerations of wide-angle incidence. Figure 4 illustrates the impact of incident CP light from varying angles of incidence and azimuth on the CD effect in metasurface structures. Figure 4a shows that the CD bandwidth remains consistent within the incidence angle range of 0° to 80°. Furthermore, Fig. 4b presents an analysis of different azimuthal incidences on CD, revealing a stable CD bandwidth within the azimuthal angle range of 0° to 90°. The observed angular stability is attributed to the diminutive dielectric thickness and unit cell size. Given the potential for incoming waves to possess arbitrary incidence angles in practical scenarios, the insensitivity to azimuth and incidence angles renders the proposed metasurface a promising candidate for diverse applications.

### Linear dichroism

**Figure 5:**
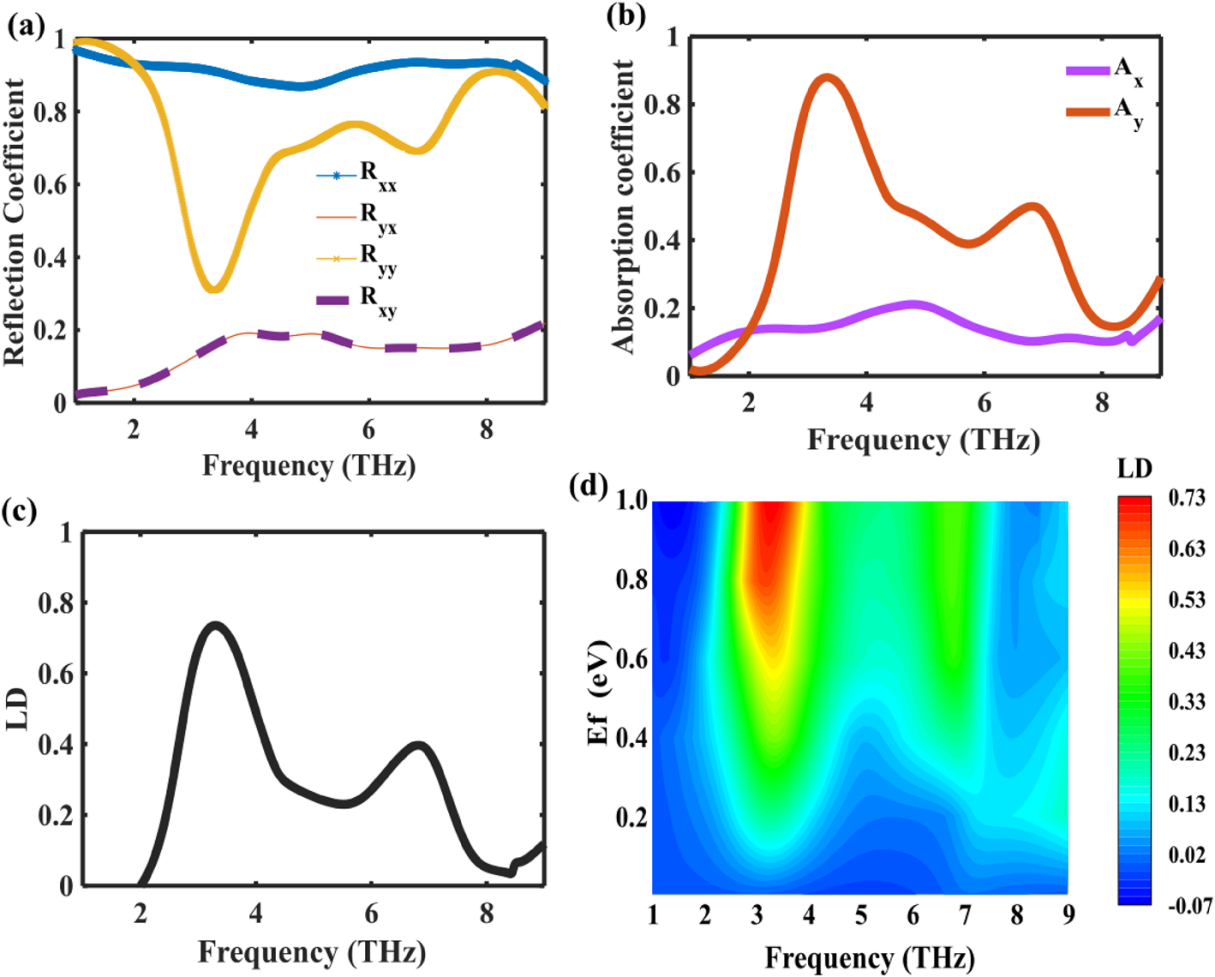
(**a**) Reflectance properties of the graphene-based metasurface with a Fermi level E_f_ of 1 eV under the excitations of LP normally incident wave. (**b**) Absorption characteristics of the proposed design under a normally incident wave. (**c**) Spectral representation of LD in the proposed design. (**d**) LD variations in the proposed structure for different chemical potentials.

In addition to showcasing CD, the proposed design also exhibits significant LD across a wideband range as shown in Fig. 5. Linear dichroism (LD) characterizes the variation in absorption between *x*-polarized and *y*-polarized light in a metasurface and is conventionally quantified as:

10 $$\mathrm{LD }= {{\text{A}}}_{{\text{y}}} - {{\text{A}}}_{{\text{x}}}$$

where ${A}_{y}$ and ${A}_{x}$ is the absorbance of a structure for *x* and *y* polarized light can be expressed as:

$${A}_{x} = 1 - {R}_{xx} - {R}_{yx}$$

$${A}_{y} = 1 - {R}_{yy} - {R}_{xy}$$

Figure 5a illustrates the simulated LP reflection coefficients (${R}_{xx}$, ${R}_{yy}$, ${R}_{xy}$, ${R}_{yx}$) of the proposed structure for linear incident THz waves.

At the resonance frequency range of 2.9–3.7 THz, the simulated co-polarization component of the reflected LP wave attains a maximum ${R}_{xx}$ value of 0.92, while the values of ${R}_{xy}$ and ${R}_{yx}$ remain below 0.2 at the same frequency points. Furthermore, Fig. 5b demonstrates that the maximum peak absorption of the TE wave reaches 88% at 3.3 THz, while the minimum absorption of 14% is observed at the same frequency for the TM incident wave. As a result, the calculated LD amounts to 74%, showcasing more than 60% absorption across the wideband range of 2.9–3.8 THz, as depicted in Fig. 5c. Consequently, the proposed device can be effectively regarded as a linear polarizer, exhibiting exceptional performance.

Furthermore, we compute the appropriate LD curves for various graphene chemical potentials, as depicted in Fig. 5d. The calculation findings indicate that LD can continuously vary from 0 to its maximum value when the graphene chemical potential increases from 0.01 to 1 eV. However, there is a little redshift in the peak frequency.

**Figure 6:**
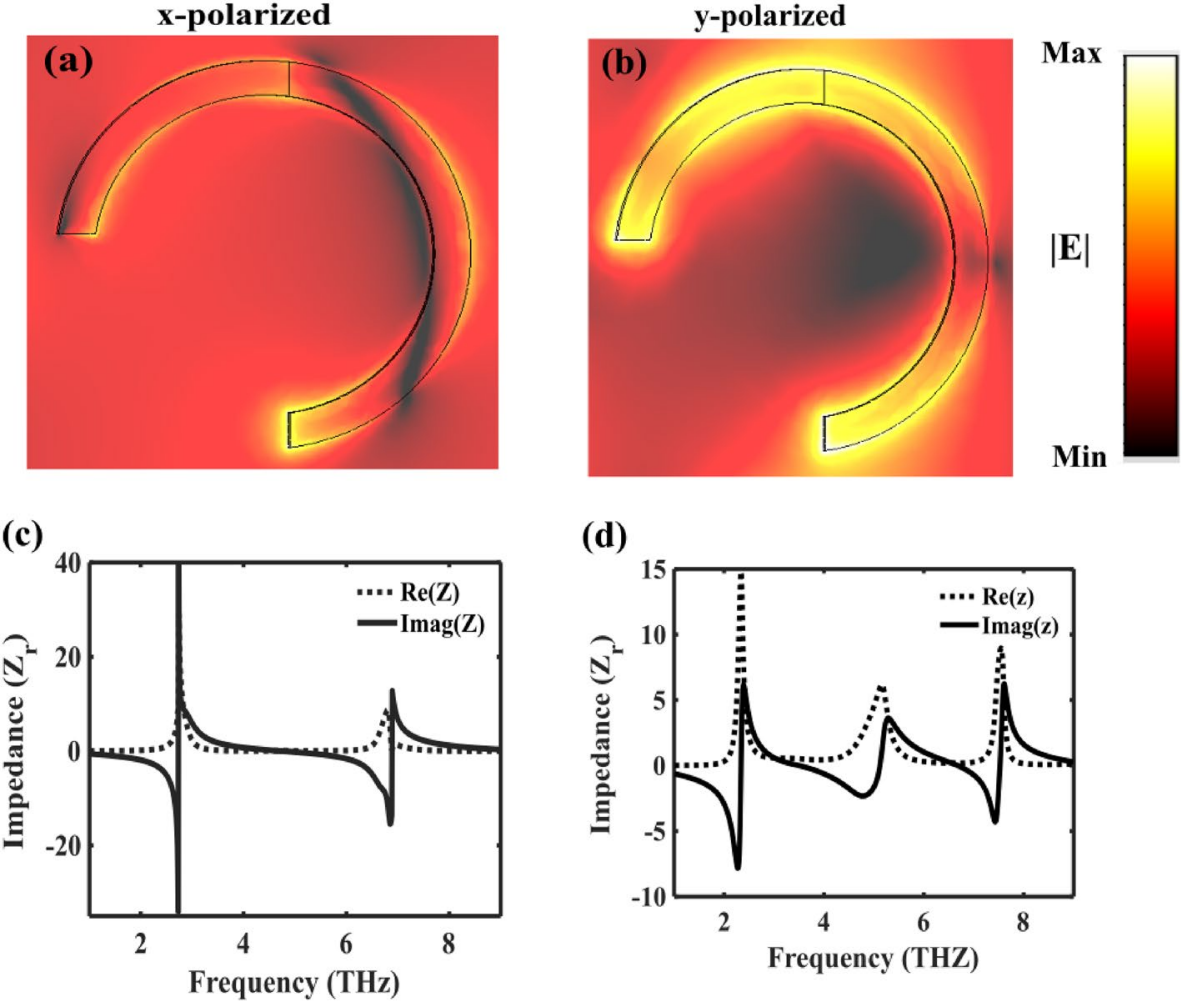
Illustrates the absolute distribution of the electric field at the peak frequency of 3.3 THz corresponding to the incident (**a**) *x*-polarized wave and (**b**) *y*-polarized wave. Additionally, it presents the real and imaginary parts of the impedance under (**c**) *x*-polarized and (**d**) *y*-polarized incidences, respectively.

To elucidate the intricate mechanisms governing the efficient generation of LD on metasurface, we present the absolute distribution |E| of the electric field and the impedance, as illustrated in Fig. 6. As evidenced in Fig. 6a and b, the metasurface exhibits notably stronger coupling to *y*-polarized light at 3.3 THz compared to *x*-polarized light, facilitating effective absorption of *y*-polarized light and reflection of *x*-polarized light.

Based on the effective medium theory, the retrieval of the effective relative impedance ${Z}_{r}$ from the reflection coefficient is articulated by the expression^37,44,45^:

11 $${Z}_{r}=\frac{Z}{{Z}_{0}}=\sqrt{\frac{{\left(1+{S}_{11}\right)}^{2}-{\left({S}_{21}\right)}^{2}}{{\left(1-{S}_{11}\right)}^{2}-{\left({S}_{21}\right)}^{2}}}$$

Here, $Z$ and ${Z}_{0}$​ represent the absorber's effective impedance and free space impedance, respectively. Consistent with impedance matching theory, aligning the effective impedance of the metasurface with that of free space aids in minimizing reflection and achieving near-perfect absorption^53^. The impedance of the metasurface under *x*-polarized incidence, depicted in Fig. 6c, reveals a lack of alignment with that of free space, resulting in diminished absorption. Conversely, the real and imaginary parts of the effective relative impedance under *y*-polarized incidence closely approximate 1 and 0, respectively, at 3.3 THz, as delineated in Fig. 6d. This alignment signifies impedance matching with free space, facilitating the attainment of nearly perfect absorption.

**Figure 7:**
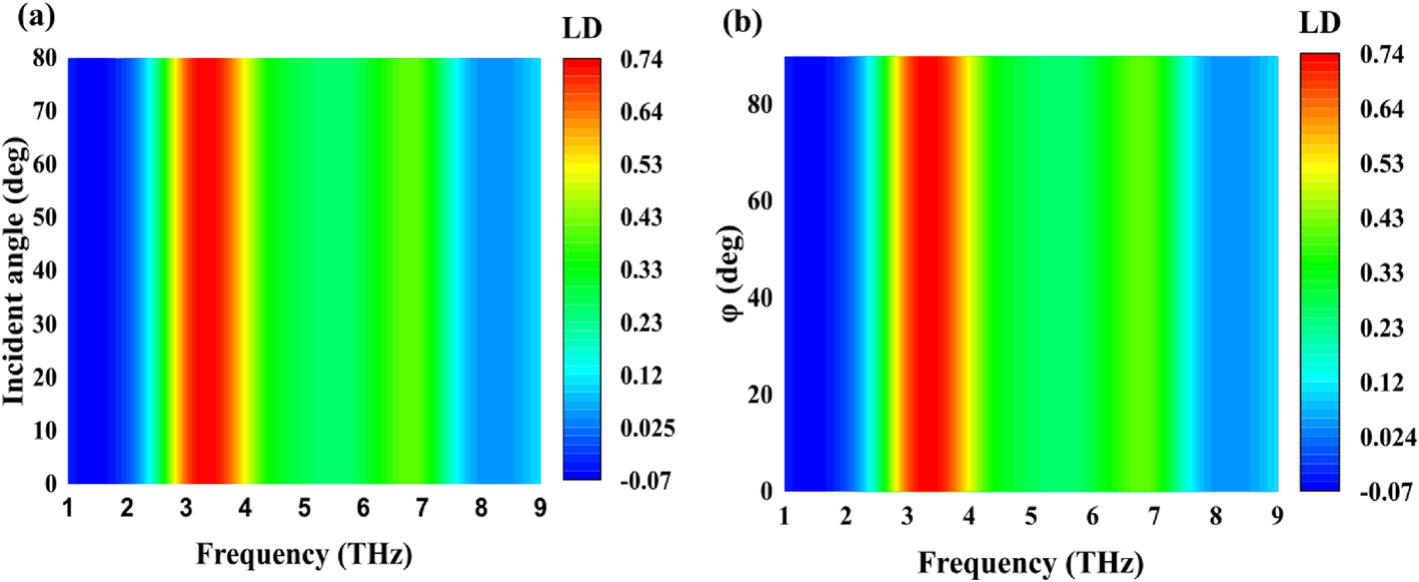
(**a**) LD of the SRR structure at different incident angle (**b**) Simulated LD at various polarization angles.

Moreover, computational analyses were conducted to ascertain the dependence of LD reflection on incident and azimuth angles. As depicted in Fig. 7a, the LD bandwidth (2.9–3.8 THz) exhibits stability within the range of incidence angles from 0° to 80°. Additionally, in Fig. 7b, the impact of various azimuthal incidences on LD is computed, demonstrating consistent LD bandwidth (2.9–3.8 THz) stability within the azimuthal angle range of 0° to 90°. In practical scenarios, arbitrary incidence angles are standard, making it necessary to consider the sensitivity of designs to both azimuth and incidence angles. Our proposed design is highly suitable for spin detectors due to its insensitivity to polarization and incident angles. This insensitivity is attributed to the symmetrical pattern of the graphene–metal split ring resonant layer at 45° in the $xy$ plane. The unique C-shaped geometric configuration, smaller unit cell size, and reduced dielectric thickness contribute to the design's angular stability. Our approach aimed to maximize the robustness of the structure's response to variations in incidence angles by maintaining a lower unit cell size and dielectric thickness and adopting a distinctive unit cell geometry.

### Polarization conversion

**Figure 8:**
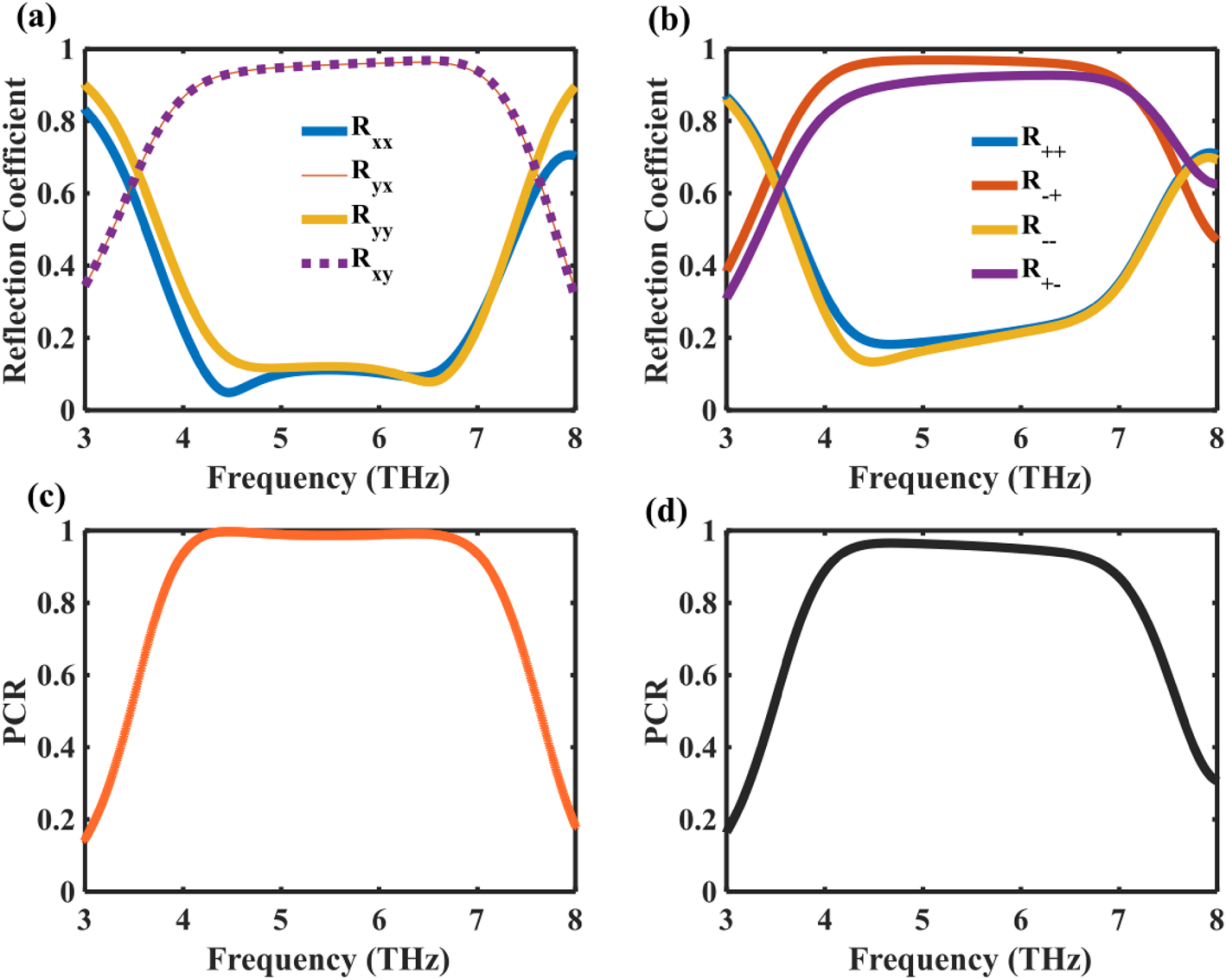
(**a**) Reflection coefficients of co- and cross-polarized configurations for a LP incident wave. (**b**) Reflection coefficients of co- and cross-polarized configurations for a CP incident wave. (**c**) PCR for a LP incident wave. (**d**) PCR for a CP incident wave.

In addition to CD and LD, the envisaged design has successfully realized the capability of polarization modulation. Figure 8a and b illustrates the simulated reflection coefficient of the unit cell for (*x/*RCP) and (*y/*LCP) polarized incidence ($\theta =15^\circ , \, \varphi =0^\circ$), in which $\left(\frac{{R}_{xx}}{{R}_{yy}}\right),\left(\frac{{R}_{++}}{{R}_{-}}\right)$ and $\left(\frac{{R}_{yx}}{{R}_{xy}}\right),\left(\frac{{R}_{-+}}{{R}_{+-}}\right)$ represent the reflection coefficients of the co-polarized and cross-polarized waves, respectively.

Figure 8a and b demonstrate that the cross-polarized reflection coefficients ($\left|{R}_{yx}\right|,\left|{R}_{xy}\right|,\left|{{\text{R}}}_{-+}\right| ,\left|{{\text{R}}}_{+-}\right|)$ consistently surpass 0.93, while the co-polarized reflection coefficients $(\left|{R}_{xx}\right|,\left|{R}_{yy}\right|,\left|{{\text{R}}}_{++}\right|,\left|{{\text{R}}}_{-}\right|$|) for linear incident waves are less than 0.1, and for circular incident waves, they are less than 0.25 within the 4.1–7.1 THz frequency range. The discerned phenomenon underscores the metasurface design's adept transformation of LP and CP into corresponding cross-polarizations over an ultrawideband spectrum. The extraordinary capability to attain nearly impeccable cross-polarized reflected fields across a broad frequency range for both LP and CP EM waves establishes the envisaged metasurface as an exceedingly proficient HWP. Its prowess lies in its ability to manipulate EM wave polarization states with remarkable efficacy.

The cross-polarization conversion (CPC) behavior is further characterized by quantifying the Polarization Conversion Ratio (PCR). This ratio is obtained by dividing the power reflected in the cross-polarized component by the total reflected power, expressed as ${\text{PCR}}=\frac{{\left|{R}_{cross}\right|}^{2}}{{\left|{R}_{cross}\right|}^{2}+{\left|{R}_{co}\right|}^{2}}$. The PCR outcomes, as illustrated in Fig. 8c and d, reveal that within the CPC bands spanning from 4.1 to 7.1 THz, the PCR consistently exceeds 95% and reaches 100% at the ultrawideband of 4.3–6.5 THz for linear incident waves, while achieving over 90% for circular incidents in the 4–6.8 THz frequency range.

**Figure 9:**
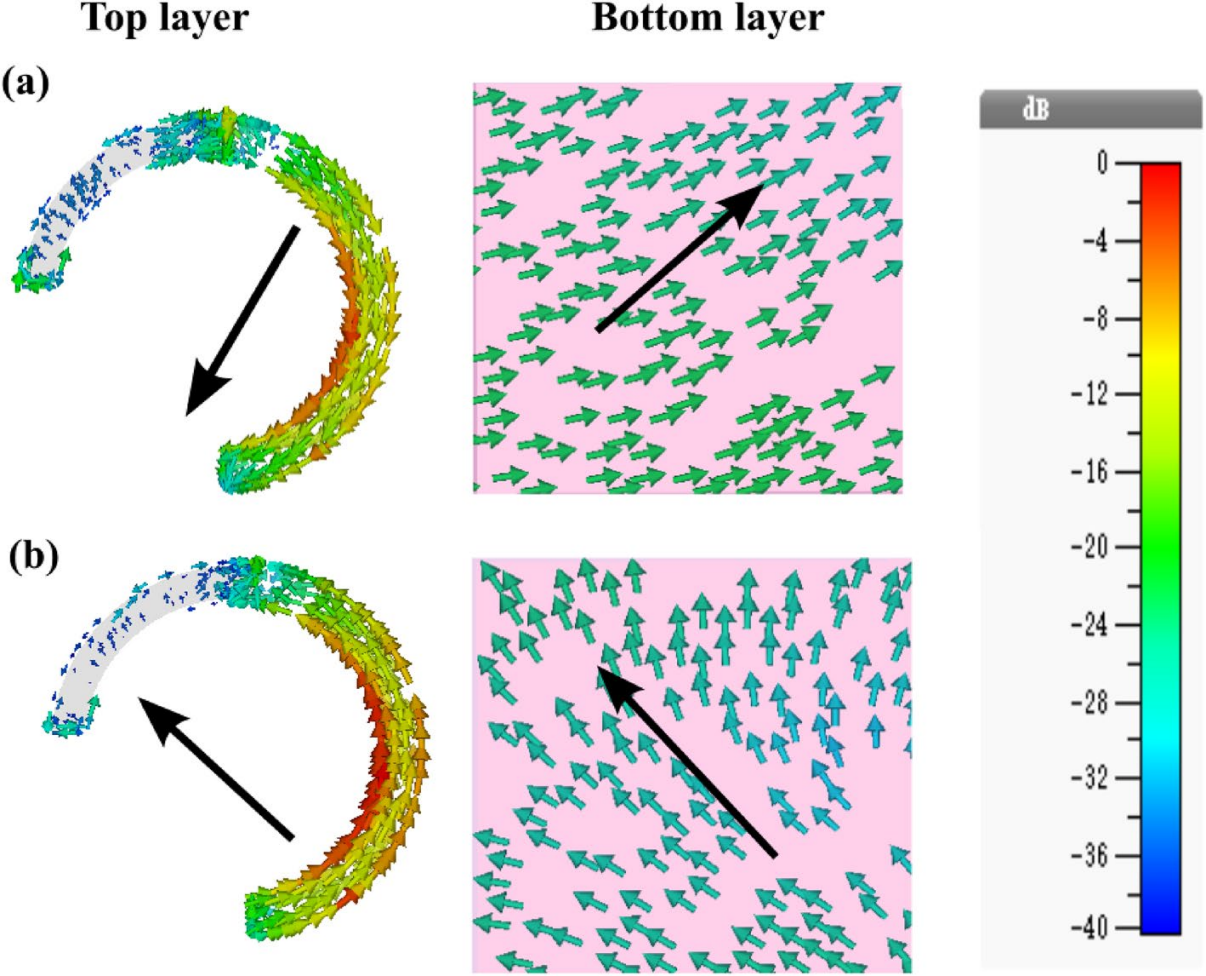
Surface current distribution for a CP incident wave at the top and bottom layer at (**a**) 4.4 THz and (**b**) 6.4 THz.

To comprehend the underlying physical mechanisms of the proposed polarization converter, we conducted simulations to examine the surface current distributions on the top and bottom layer of proposed SRR at resonant frequencies of 4.4 THz and 6.4 THz, respectively, as depicted in Fig. 9. The observed surface currents along the SRR resonator are antiparallel to those on the back metallic sheet at 4.4 THz, indicating the formation of current loops in the intermediate dielectric layer, a characteristic known as magnetic resonance (Fig. 9a). Conversely, at 6.4 THz, the surface currents along the top SRR parallel those on the back metallic layer, corresponding to electric resonance (Fig. 9b). Magnetic and electric dual resonances are pivotal in achieving high efficiency and wide bandwidth. Ultra-wide bandwidth enhancement arises from the superposition of multiple PCR peaks around resonance frequencies.

The comparative analysis of the proposed metasurfaces performance against previously reported designs is presented in Table 1. Upon scrutiny of the data in Table 1, it is evident that most contemporary multifunctional devices are confined to 1–2 functions, encountering challenges such as complex structures, non-tunability, and diminished efficiency. We advocate for developing a terahertz frequency band three-function tunable metasurface in response to these limitations. Theoretical calculations substantiate that the proposed metasurface excels in supporting wideband tunable CD, LD, and the conversion of linear and circular polarizations to their respective cross-polarizations across a broad spectrum.

**Table 1:** Comparison of pre-existing designs.

References	$C{D}_{max}$ and bandwidth	$L{D}_{max}$ and bandwidth	CPC bandwidth	Tunability	Angular stability
^10^	91% (12.3–12.4 THz)	–	–	–	Yes up-to 30°
^22^	–	–	(0.4–1.9 THz)	Yes	–
^45^	42% (1.1 THz)	–	–	Yes	Yes up-to 30°
^52^	90% (22 THz)	70% (22 THz)	–	–	–
^53^	81% (1.0 THz)	89% (1.16 THz)	–	Yes	Yes up-to 30°
Proposed work	75% (3.1–7.4 THz)	74% (2.9–3.8 THz)	(4.1–7.1 THz)	YES	Yes up-to 80° and azimuth angle up-to 90°

Furthermore, many reported CDs and LDs lack tunability and operate exclusively under normal or oblique incidence conditions. Consequently, our work represents a notable advancement in integrating photonic devices in the terahertz domain. Notably, in contrast to specific currently reported multi-layered devices, our proposed SRR metasurface adopts the simplest sandwich reflection structure, enhancing practicality in manufacturing and integration processes.

### Potential fabrication process

Despite the simulation calculations performed in this study, it is essential to consider the practical feasibility of the proposed structure's manufacturing. Firstly, a chemical vapor phase precipitation method is utilized to grow an 11-um-thick Topas medium on top of an underlying gold film. A supporting layer of PMMA (poly(methyl methacrylate), C2, Microchem) is employed for the transfer of graphene. The transferred graphene is then patterned using ultraviolet (UV) lithography. Subsequently, the portions of graphene not covered by the resist are etched using a plasma asher^54,55^. After developing the top layer photoresist, an oxygen plasma is employed to create a ribbon-patterned graphene according to the shape of the developed photoresist. Finally, metal split rings are obtained through photolithography and metallization^56^.

## Conclusion

In summary, our study introduces a terahertz switchable metasurface employing a graphene–metal hybrid structure for multifunctionality. By exploiting the distinct conductivities of two materials, we enable the excitation of spin-selective responses to incident light, with switching between functions controlled by incident and polarization angles. Theoretical results indicate that circularly polarized light yields a CD intensity exceeding 40% (max CD 75%) within the 3.1 to 7.4 THz range. Linearly polarized light results in LD intensity surpassing 60% (max CD 74%) within the 2.9 to 3.8 THz range. The metasurface achieves 100% PCR in converting linear and circular polarizations in the 4.1 to 7.1 THz range. Our approach uniquely leverages phase change materials and electromagnetic wave polarization angles, reducing structural complexity and integration challenges in multifunctional devices. This work advances terahertz metasurface technology, demonstrating potential applications in spin-selective light manipulation and providing a foundation for future developments in photonic device integration.

## Data Availability

The data that support the findings of this study are available upon reasonable request from the authors.

## References

[1] Ferguson B, Zhang XC (2002). Materials for terahertz science and technology. Nat. Mater..

[2] Tonouchi M (2007). Cutting-edge terahertz technology. Nat. Photonics.

[3] Xu W, Xie L, Ying Y (2017). Mechanisms and applications of terahertz metamaterial sensing: A review. Nanoscale.

[4] Islam M, Bhardwaj A, Bhowmik BK, Kumar G (2023). Terahertz meta-waveguide based upon strongly near-field coupled split-ring resonators. J. Infrared Millim. Terahertz Waves.

[5] Islam M, Bhowmik BK, Dhriti KM, Mohan D, Ahmad A, Kumar G (2022). Thin film sensing in a planar terahertz meta-waveguide. J. Opt..

[6] Chen HT, Padilla WJ, Zide JM, Gossard AC, Taylor AJ, Averitt RD (2006). Active terahertz metamaterial devices. Nature.

[7] Landy NI, Sajuyigbe S, Mock JJ, Smith DR, Padilla WJ (2008). Perfect metamaterial absorber. Phys. Rev. Lett..

[8] Qureshi UUR, Khan MI, Hu B (2022). A theoretical proposal for an actively controlled ultra-wideband absorber based on vanadium dioxide hybrid metamaterials. Appl. Sci..

[9] Zhang W, Wang Y, Wen X, Zhang Z (2015). Giant circular dichroism induced by silver nanocuboid heterodimers. Appl. Opt..

[10] Qureshi, U. U. R., Khan, M. I., & Hu, B. Realizing efficient THz circular dichroism using ultra-thin chiral metasurface. *Physica Scripta*. (2023)

[11] Qureshi, U. U. R., Basir, S., Ahmad, M., Jalal, A., Iqbal, R. & Khan, M. I. Realizing high-efficient multifunctional and tunable multiband THz metasurface for polarization and phase modulation. *Optik*, 171663 (2024).

[12] Tang, P., Xu, J. & Wang, R. K. Imaging and visualization of the polarization state of the probing beam in polarization-sensitive optical coherence tomography. *Appl. Phys. Lett.* **113**(23) (2018).

[13] Qureshi UUR, Hu B, Basir S, Ahmad M, Jalal A, Khan MI (2023). Design and experimental realization of multifunctional anisotropic metasurface for efficient polarization manipulation in microwave frequencies. Physica Scripta.

[14] Bao Z, Wang J, Hu ZD, Chen Y, Zhang C, Zhang F (2021). Coordination multi-band absorbers with patterned irrelevant graphene patches based on multi-layer film structures. J. Phys. D Appl. Phys..

[15] Hu J, Qing Y, Yang S, Ren Y, Wu X, Gao W, Wu C (2017). Tailoring total absorption in a graphene monolayer covered subwavelength multilayer dielectric grating structure at near-infrared frequencies. JOSA B.

[16] Ai H, Kang Q, Wang W, Guo K, Guo Z (2021). Multi-beam steering for 6G communications based on graphene metasurfaces. Sensors.

[17] Asgari S, Granpayeh N (2019). Tunable mid-infrared refractive index sensor composed of asymmetric double graphene layers. IEEE Sens. J..

[18] Liang C, Niu G, Chen X, Zhou Z, Yi Z, Ye X, Xiao S (2019). Tunable triple-band graphene refractive index sensor with good angle-polarization tolerance. Opt. Commun..

[19] Scidà A, Haque S, Treossi E, Robinson A, Smerzi S, Ravesi S, Palermo V (2018). Application of graphene-based flexible antennas in consumer electronic devices. Mater. Today.

[20] Wan Y, An Y, Tao Z, Deng L (2018). Manipulation of surface plasmon resonance of a graphene-based Au aperture antenna in visible and near-infrared regions. Opt. Commun..

[21] Asgari S, Granpayeh N (2017). Tunable plasmonic dual wavelength multi/demultiplexer based on graphene sheets and cylindrical resonator. Opt. Commun..

[22] Chen D, Yang J, Huang J, Bai W, Zhang J, Zhang Z, Xie W (2019). The novel graphene metasurfaces based on split-ring resonators for tunable polarization switching and beam steering at terahertz frequencies. Carbon.

[23] Zhang K, Liu Y, Li S, Xia F, Kong W (2021). Actively tunable bi-functional metamirror in a terahertz band. Opt. Lett..

[24] Qureshi, U. U. R., Hu, B., Ahmad, M. & Jalal, A. Graphene-based triple-band tunable metasurface with strong circular dichroism for Hz communication. *IEEE Photonics Technol. Lett.* (2023).

[25] Lv TT, Li YX, Ma HF, Zhu Z, Li ZP, Guan CY, Cui TJ (2016). Hybrid metamaterial switching for manipulating chirality based on VO2 phase transition. Sci. Rep..

[26] Jing, L., Wang, Z., Yang, Y., Zheng, B., Liu, Y., & Chen, H. (2017). Chiral metamirrors for broadband spin-selective absorption. *Appl. Phys. Lett*, **110**(23).

[27] Gruev V, Perkins R, York T (2010). CCD polarization imaging sensor with aluminum nanowire optical filters. Opt. Express.

[28] Li J, Li Z, Deng L, Dai Q, Fu R, Deng J, Zheng G (2020). Dichroic polarizing metasurfaces for color control and pseudo-color encoding. IEEE Photonics Technol. Lett..

[29] Amin M, Siddiqui O, Farhat M (2020). Linear and circular dichroism in graphene-based reflectors for polarization control. Phys. Rev. Appl..

[30] Valev VK, Baumberg JJ, Sibilia C, Verbiest T (2013). Chirality and chiroptical effects in plasmonic nanostructures: Fundamentals, recent progress, and outlook. Adv. Mater..

[31] Plum, E. (2016). Extrinsic chirality: Tunable optically active reflectors and perfect absorbers. *Appl. Phys. Lett.* **108**(24).

[32] Qureshi UUR, Basir S (2024). Dual-functional multiband metasurface for efficient circular and linear dichroism. Opt. Mater..

[33] Cao T, Wei CW, Mao LB, Wang S (2015). Tuning of giant 2D-chiroptical response using achiral metasurface integrated with graphene. Opt. Express.

[34] Cui Y, Fung KH, Xu J, Ma H, Jin Y, He S, Fang NX (2012). Ultrabroadband light absorption by a sawtooth anisotropic metamaterial slab. Nano Letters.

[35] Plum E, Liu XX, Fedotov VA, Chen Y, Tsai DP, Zheludev NI (2009). Metamaterials: optical activity without chirality. Phys. Rev. Lett..

[36] Wu J, Zhou C, Yu J, Cao H, Li S, Jia W (2014). TE polarization selective absorber based on metal-dielectric grating structure for infrared frequencies. Opt. Commun..

[37] Chen Y, Yang X, Gao J (2019). 3D Janus plasmonic helical nanoapertures for polarization-encrypted data storage. Light Sci. Appl..

[38] Wang SY, Liu W, Geyi W (2018). A circular polarization converter based on in-linked loop antenna frequency selective surface. Appl. Phys. B.

[39] Serebryannikov AE, Lakhtakia A, Vandenbosch GA, Ozbay E (2022). Transmissive terahertz metasurfaces with vanadium dioxide split-rings and grids for switchable asymmetric polarization manipulation. Sci. Rep..

[40] Nguyen TM, Vu DL, Nguyen TQH, Kim JM (2022). Reconfigurable broadband metasurfaces with nearly perfect absorption and high efficiency polarization conversion in THz range. Sci. Rep..

[41] Xie, Q., Sun, J., Su, C., Xia, F., Wang, M., Zhang, K. & Yun, M. (2023). Multifunctional metasurface for broadband absorption and polarization conversion based on graphene-VO2. *Diamond Relat. Mater.*, 110119.

[42] Wen D, Chen S, Yue F, Chan K, Chen M, Ardron M, Chen X (2016). Metasurface device with helicity-dependent functionality. Adv. Opt. Mater..

[43] Bai Y, Ouyang C, Zhang S, Yao Z, Liu K, Liu S, Tian Z (2023). Ge_2_Sb_2_Te_5_-based efficient switching between a cross-polarization conversion and a circular-to-linear polarization conversion. Opt. Lett..

[44] Ghosh SK, Das S, Bhattacharyya S (2022). Graphene-based metasurface for tunable absorption and transmission characteristics in the near mid-infrared region. IEEE Trans. Antennas Propag..

[45] Qureshi UUR, Hu B, Khan MI, Ahmad M (2024). Multifunctional active terahertz metasurface with electromagnetically induced transparency, perfect absorption, and circular dichroism. Opt. Commun..

[46] Zhu H, Zhang Y, Ye L, Li Y, Xu Y, Xu R (2020). Switchable and tunable terahertz metamaterial absorber with broadband and multi-band absorption. Opt. Express.

[47] Harada Y, Ukhtary MS, Wang M, Srinivasan SK, Hasdeo EH, Nugraha AR, Kono J (2017). Giant terahertz-wave absorption by monolayer graphene in a total internal reflection geometry. ACS Photonics.

[48] Addison Z, Park J, Mele EJ (2019). Twist, slip, and circular dichroism in bilayer graphene. Phys. Rev. B.

[49] Wang Z, Jia H, Yao K, Cai W, Chen H, Liu Y (2016). Circular dichroism metamirrors with near-perfect extinction. Acs Photonics.

[50] Wang L, Huang X, Li M, Dong J (2019). Chirality selective metamaterial absorber with dual bands. Opt. Express.

[51] Jiang H, Peng K, Cui Y, Zhong J, Zhang H, Jiang Y, Zhao W (2022). Design and simulation of a GST-based metasurface with strong and switchable circular dichroism. Opt. Lett..

[52] Tian Y, Yin Z, Zhao X, Wang J, Liu P, Zhang K, Kong W (2023). Multi-polarization selective absorber based on terahertz metasurface. Results Phys..

[53] Huang Y, Xie X, Pu M, Guo Y, Xu M, Ma X, Luo X (2020). Dual-functional metasurface toward giant linear and circular dichroism. Adv. Opt. Mater..

[54] Lee S, Baek S, Kim TT, Cho H, Lee S, Kang JH, Min B (2020). Metamaterials for enhanced optical responses and their application to active control of terahertz waves. Adv. Mater..

[55] Park, H., Jeong, S., Seo, C., Park, H., Oh, D., Shim, J. E. & Kim, T. T. (2023). Electrically tunable THz graphene metasurface wave retarders. *Nanophotonics*, (0). 10.1515/nanoph-2022-0812 PMC11501122 39633775

[56] Li X, Tang S, Ding F, Zhong S, Yang Y, Jiang T, Zhou J (2019). Switchable multifunctional terahertz metasurfaces employing vanadium dioxide. Sci. Rep..

